# Immune Infiltration of Ulcerative Colitis and Detection of the m6A Subtype

**DOI:** 10.1155/2022/7280977

**Published:** 2022-06-25

**Authors:** Chenzheng Gu, Junlu Wu, Wei Zhang, Yiwen Yao, Wenhui Yan, Yi Yuan, Weiwei Wang, Anquan Shang

**Affiliations:** ^1^Department of Laboratory Medicine, Shanghai Tongji Hospital, School of Medicine, Tongji University, Shanghai 200065, China; ^2^Department of Internal Medicine V-Pulmonology, Allergology, and Respiratory Intensive Care Medicine, Saarland University Hospital, Homburg 66424, Germany; ^3^Department of Laboratory Medicine, Yangzhi Rehabilitation Hospital (Shanghai Sunshine Rehabilitation Center), Tongji University School of Medicine, No. 2209 Guangxing Rd., Shanghai 201619, China; ^4^Department of Pathology, Tinghu People's Hospital of Yancheng City, Yancheng 224005, China

## Abstract

Ulcerative colitis (UC) is an inflammatory bowel disease characterized by persistent colon inflammation. N6-methyladenosine (m6A) methylation is one of the most prevalent RNA modifications with key roles in both normal and illness, but m6A methylation in ulcerative colitis is unknown. This research investigated m6A methylation in UC. We examined the expression of known m6A RNA methylation regulators in UC using the Gene Expression Omnibus database (GEO database). First, we used m6A regulators to examine m6A change in UC samples. These two patient groups were created by clustering three m6A gene expression datasets. These genes were then utilized to build an m6A gene network using WGCNA and PPI. These networks were built using differentially expressed genes. The 12 m6A regulators were found to be dispersed throughout the chromosome. The study's data were then connected, revealing positive or negative relationships between genes or signaling pathways. Then, PCA of the 12 m6A-regulated genes indicated that the two patient groups could be discriminated in both PC1 and PC2 dimensions. The ssGSEA algorithm found that immune invading cells could be easily distinguished across diverse patient groups. Both groups had varied levels of popular cytokines. The differential gene analysis of the two samples yielded 517 genes like FTO and RFX7. It found 9 hub genes among 121 genes in the blue module, compared their expression in two groups of samples, and found that the differences in expression of these 9 genes were highly significant. The identification of 9 possible m6A methylation-dependent gene regulatory networks suggests that m6A methylation is involved in UC pathogenesis. Nine candidate genes have been identified as possible markers for assessing UC severity and developing innovative UC targeted therapeutic approaches.

## 1. Introduction

One of a kind of inflammatory bowel disease (IBD) is ulcerative colitis (UC), a chronic inflammatory disease of the colon that most frequently affects individuals 30–40 years of age [1]. Other organs and tissues may be harmed as a result of the patient's aberrant autoimmune activity. Colon cancer is more common in those with UC. When it comes to the biology of ulcerative colitis, it is difficult to say because of the disease's multiple aetiopathogeneses. As a result, current treatment methods fail to account for the disease's complexity, heterogeneity, and unpredictability and therefore do not provide targeted, long-lasting benefits. If various UC subtypes can be identified, treatment regimens may be developed for individuals with diverse subtypes, resulting in better outcomes for those with UC.

Recent research has revealed that UC pathogenesis is influenced and functionally integrated by a person's genetic predisposition, environmental influences, gut bacteria, and immunological response [2]. In spite of this, the exact etiology of UC remains a mystery, and the majority of patients are treated with surgery and conventional treatments, which are ineffective in relieving symptoms entirely and may cause side effects that have a negative impact on a patient's quality of life [3]. There is a clear need to dig further into the pathophysiology of UC and identify novel therapeutic approaches.

The dynamic control and reversible posttranscriptional regulation of m6A have made it a hot topic among the numerous (>100) distinct chemical alterations. As a result of its interactions with many RNAs and signaling pathways, it has a significant impact on disease development. There have been a lot of advances in our understanding of mRNA's metabolism since the discovery of m6A in the early 1970s [4], and it is now the most common alteration identified in both bacteria and plants. M6A methylation seems to be important throughout embryonic development, circadian rhythm, the cell cycle, and cancer [5]. A growing body of data suggests that the posttranscriptional gene regulatory mechanism m6A methylation is involved in the development of IBD. As an example, in mice, the deletion of an enzyme known as 14 (METTL14), which is part of the RNA methyltransferase complex, causes spontaneous colitis and a Th1/Th17 phenotype. Colitis develops as a result of faulty regulatory T cells and as a result of the microbiota in the gut [6]. It is been shown in another research that mice with Foxp3-mediated deletion of the methyltransferase like 3 (METTL3) in regulatory T cells produce an extreme immunological response, which is typical of intestinal inflammation [7]. M6A alteration is examined in this article in relation to intestinal mucosal immunity, dendritic cell (DC) and T cell regulation, and its increasing clinical relevance in IBD and CRC [8].

We used the public Gene Expression Omnibus database (GEO database) to comprehensively examine the expression of commonly reported m6A RNA methylation regulators in UC. First, using 12 m6A regulators, we looked at the pattern of m6A alteration in UC samples. Three datasets of m6A gene expression patterns were clustered to produce two patient groups, A and B. These genes were then used to create an m6A-related gene network, which included the WGCNA as well as a protein-protein interaction network (PPI). The differentially expressed genes were then used to construct these networks. In the end, we found nine hub genes. Future biological study will be guided by this hub gene.

## 2. Methods

### 2.1. Data Sources and Study Selection

We searched the public GEO database for all expression microarrays that matched UC keywords (https://www.ncbi.nlm.nih.gov/geo/query/acc.cgi?acc) [9]. Data from the GSE75214, GSE87473, and GSE109142 datasets were analyzed after unnecessary information was removed.

### 2.2. Data Preprocessing

The expression profiles of GSE75214, GSE87473, and GSE109142 were combined to preserve data consistency, and the estimated precision weights of each observation were multiplied by the matching log2 to provide final gene expression levels. The “sva” *R* package was used to do batch correction. Dimension reduction and visual analysis are performed using the *R* package “tsne.”

### 2.3. Selecting m6A RNA Methylation Regulators via Systematic Review

The PubMed databases were systematically searched from conception to September 28, 2021, for all relevant English language research. Two reviewers (F Liu and ZS Wu) separately conducted a manual search of the chosen papers and pertinent review articles. For this study, only genes proven to be methylation regulators of the m6A RNA methylation complex in animals or cells will be eligible. These genes include KIAA1429, METTL3, METTL14, RBM15, WTAP, HNRNPC, YTHDC1, YTHDC2, YTHDF1, YTHDF2, ALKBH5, and FTO [10–13].

### 2.4. Unsupervised Clustering of 12 m6A Regulators

The expression profiles of 12 m6A genes were used in an unsupervised cluster analysis to discover distinct m6A expression patterns, and the patients were then categorized and grouped for further study. The number of clusters and their stability was determined using a consensus clustering method. Clustering is done using the “ConsensusClusterPlus” *R* package and the Euclidean distance, which is performed 1000 times to ensure classification stability.

### 2.5. m6A Induced Molecular Subtypes of UC

In order to consistently cluster and select m6A subtypes from prognostic m6A expression profiles, we used consensus *k*-means clustering. In order to achieve 80% coverage, 100 rounds of clustering were done. Using the consensus matrix heatmaps, CDF curves of the consensus score, unambiguous separation of the heatmaps, features of the consensus CDF plots, and sufficient pair-wise consensus values between cluster members, the optimum cluster number was found.

### 2.6. Gene Set Variation Analysis (GSVA)

In order to assess changes in route and biological process activity in samples, scientists utilize the GSVA nonparametric, unsupervised approach. The *R* package “GSVA” was used to conduct GSVA enrichment analysis on the various m6A gene expression patterns to examine the variations in biological processes. The MSIGDB.v7.1. symbol gene set was downloaded from the MSigDB database for GSVA analysis GSEA (https://www.gsea-msigdb.org/gsea/index.jsp).

### 2.7. Analysis of Differentially Expression Genes in Different Clusters

The expression profiles of the three datasets were integrated, and the differentially expressed genes among different groups were analyzed by *R* package “limma.” To screen out the differentially expression genes (DEGs), we set adjusted *p* values <0.01 and |log_2_^(foldchange)^| > 0.5 as cut-off criteria.

### 2.8. Assessment for Immune Infiltration among Subtypes

Furthermore, we evaluated the variation of immune status among subtypes. Gene Set Single sample Gene Set Enrichment Analysis (ssGSEA) is an extension of the GSEA method to assess cell infiltration in the tumor microenvironment of samples [14]. Each GSEA ES represents the degree to which the genes in a particular gene set are coordinately up- or downregulated within a sample. Immune cells included immune enhancing cells (Th1 cells, T cells CD4 activated, NK cells activated, and B cells activated) and immune suppressive cells (Th2 cells and Treg cells). Differential expression analysis using moderated *t*-tests would be utilized to assess the expression distribution of proinflammatory cytokines and enrichment of immune cells among subgroups, and *p* < 0.05 was recognized as significant results. Proinflammatory cytokines included interleukin-1*β* (IL-1*β*), interleukin-6 (IL-6), and tumor necrosis factor (TNF).

### 2.9. Assessing the Heterogeneity of Biological Function among Subtypes

WGCNA is a system biology method used to describe gene association patterns between different samples. It can be used to identify highly covarying gene sets and to identify candidate biomarker genes or therapeutic targets based on the interconnectedness of gene sets and the association between gene sets and phenotypes. We attempted to find gene sets which significantly correlated to m6A subtypes through WGCNA, using WGCNA *R* package to determine coexpressed genes using all expressed genes in microarrays [15]. Module eigengene (ME) was calculated as the first principal component of gene expression for the module and interrelatedness of each module by eigengene network clustering. MEs were compared with m6A subtype information using Spearman's correlation corrected for subtypes, and *p* values were adjusted for multiple comparisons by false discovery rate. Genes from modules which highly associated with m6A subtypes (the maximum correlation coefficient and *p* < 0.05) were selected for further Gene Ontology (GO) analysis.

Then, PPI interaction network and hub gene screening were constructed by key module genes in WGCNA network. Protein-protein interaction network was based on STRING website (https://string-db.org/). The hub genes were extracted from Cytoscape. The intersection of hub genes extracted through WGCNA and PPI network was taken, and the *K*-means was used to perform cluster analysis on the expression profile data of hub genes.

### 2.10. Software and Versions

Rx64 (version 3.6.1) was conducted to run and process data scripts, output results, and plot diagrams; Cytoscape (version 3.6.1) was performed to plot network diagrams.

## 3. Results

### 3.1. m6A-Cluster Subtypes and Correlation Analysis in UC

To begin, we chose a total of 12 m6A RNA methylation regulators from the literature and the genes in our three GEO datasets with accessible RNA expression data for our study (Table 1). There were m6A writers, readers, and erasers on this group of regulators. KIAA1429, METTL3, METTL14, RBM15, and WTAP have all been identified as m6A writers. HNRNPC, YTHDC1, YTHDC2, YTHDF1, and YTHDF2 were also among the m6A readers [16]. Erasers in the m6A family included the ALKBH5 and FTO.

### 3.2. The Chromosome Distribution of Factor m6A

**Figure 1:**
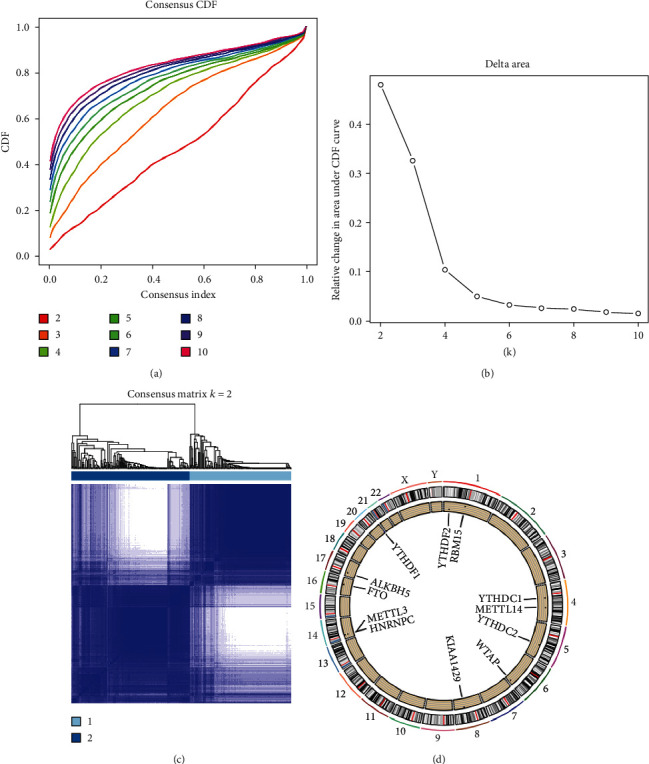
Identification of UC subtypes based on the m6A RNA methylation regulators. (a) The cumulative distribution function (CDF) curves is the integral of probability density function, which can completely describe the probability distribution of a real random variable, and established using consensus clustering approach. CDF curves of consensus scores based on different subtype number (*k* = 2, 3, 4, 5, 6, 7, 8, 9, 10), and the corresponding color are represented. (b) The CDF delta area curve of all samples when *k* = 2. (c) Consensus heatmaps for *k* = 2. (d) The location of the m6A gene on the chromosome is shown by Circos.

Unsupervised cluster analysis of the expression patterns of 12 m6A RNA methylation regulators was conducted when clustering stability increased from 2 to 10 in cohort (Figure 1(a)). A reasonable choice for *k* = 2 appeared to be the comparable expression of the m6A-RNA-methylation regulators (Figures 1(b) and 1(c)). It was shown that there are two distinct subgroups of UC patients as a consequence (cluster A and cluster B). The chromosomal distribution of 12 m6A regulators was also examined, and these 12 genes were shown to be widely distributed (Figure 1(d)).

### 3.3. The m6A Correlation Analysis and PCA Analysis

**Figure 2:**
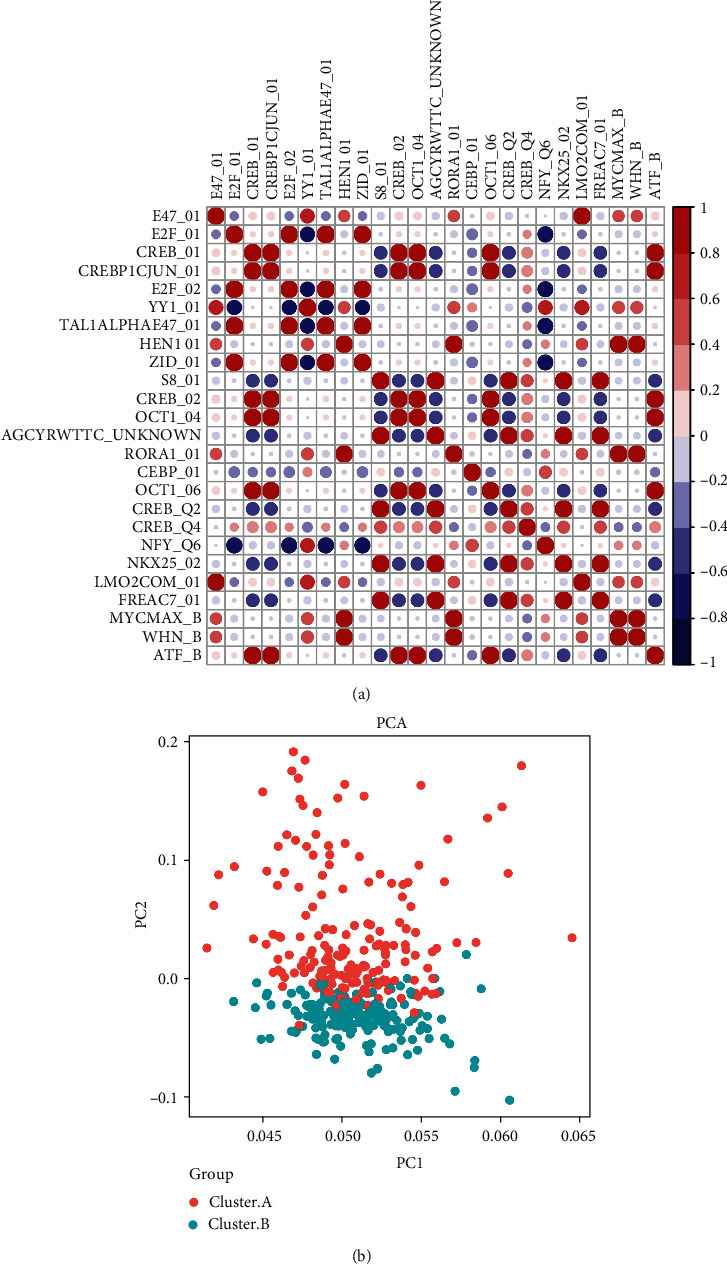
m6A correlation analysis and PCA analysis. (a) GSVA correlation analysis results. (b) PCA analysis results.

GSVA enrichment was used to examine the expression patterns of 12 m6A genes. The MSIGDB.v7.1. gene set symbols were obtained from the MSigDB database and saved locally. On the basis of the correlation study, several genes or signaling pathways had positive or negative relationships (Figure 2(a)). Further PCA analysis of the expression profiles of 12 m6A regulators was performed, and the Figure 2(b) shows that patient samples from clusters A and B could be differentiated in PC1 and PC2 dimensions essentially (Figure 2(b)).

### 3.4. Immune Status Heterogeneity of m6A-Cluster Subtypes

**Figure 3:**
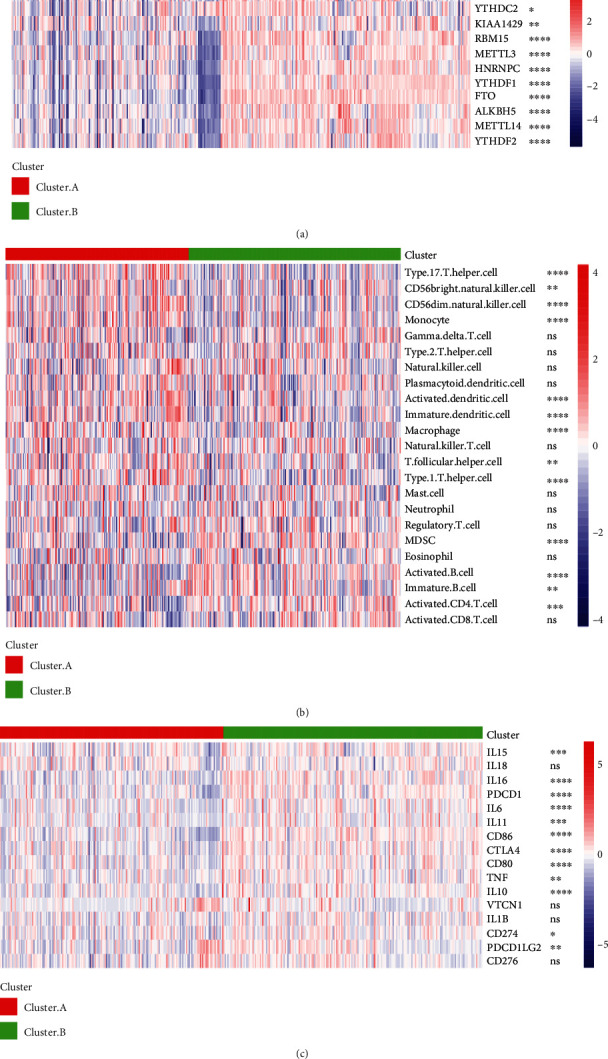
Differences of m6A expression, immune cell infiltration, and cytokines were displayed among m6A-cluster subtypes. (a) Differences of 12 m6A regulators expression levels among different m6A-cluster subtypes. (b, c) Differences in immune cell infiltration and cytokines between different m6A-cluster subtypes. ns, *p* > 0.05; ^∗^*p* < 0.05; ^∗∗^*p* < 0.01; ^∗∗∗^*p* < 0.01, *p* < 0.001; ^∗∗∗∗^*p* < 0.0001.

Inflammation of the intestinal mucosal barrier and an unusually long-lasting immune response are both recognized complications of IBD. As a result, m6A-cluster subtype immune cell infiltration must be characterized. In cluster A and cluster B, the expression patterns of m6A regulators were shown on heat maps, and the two gene groups were clearly separated (Figure 3(a)). The ssGSEA algorithm was then used to estimate the degree of immune cell infiltration in the tumor microenvironment in the samples. The gene set of different types of immune cells was obtained from Charoentong's study, and this gene set contains various human immune cells such as activated CD8 T cells, activated DC cells, macrophages, NK cells, regulatory T cells, and 24 other different cell types. The ssGSEA algorithm enriches the score to represent the abundance of immune infiltrating cells in the tumor microenvironment in each sample. The score was also used to assess the risk of the 24 different types of immune infiltrating cells. The heat map results showed that immune infiltrating cells were clearly differentiated between different patient subgroups Cluster.A and Cluster.B (Figure 3(b)). The expression of popular cytokines also differed significantly between Cluster.A and Cluster.B. For example, IL16 and PDCD1 were significantly more expressed in Cluster.B samples than in Cluster.A samples (Figure 3(c)).

### 3.5. Heterogeneity of Other Biological Function of m6A-Cluster Subtypes

**Figure 4:**
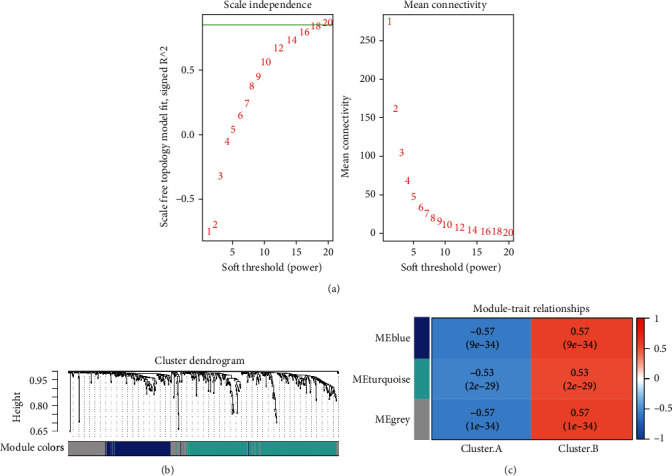
Weighted gene coexpression network analysis. (a) Analysis of network topology for various soft thresholding powers (soft = 20). (b) Clustering effect of different coexpression network modules. (c) Module-trait relationship.

**Figure 5:**
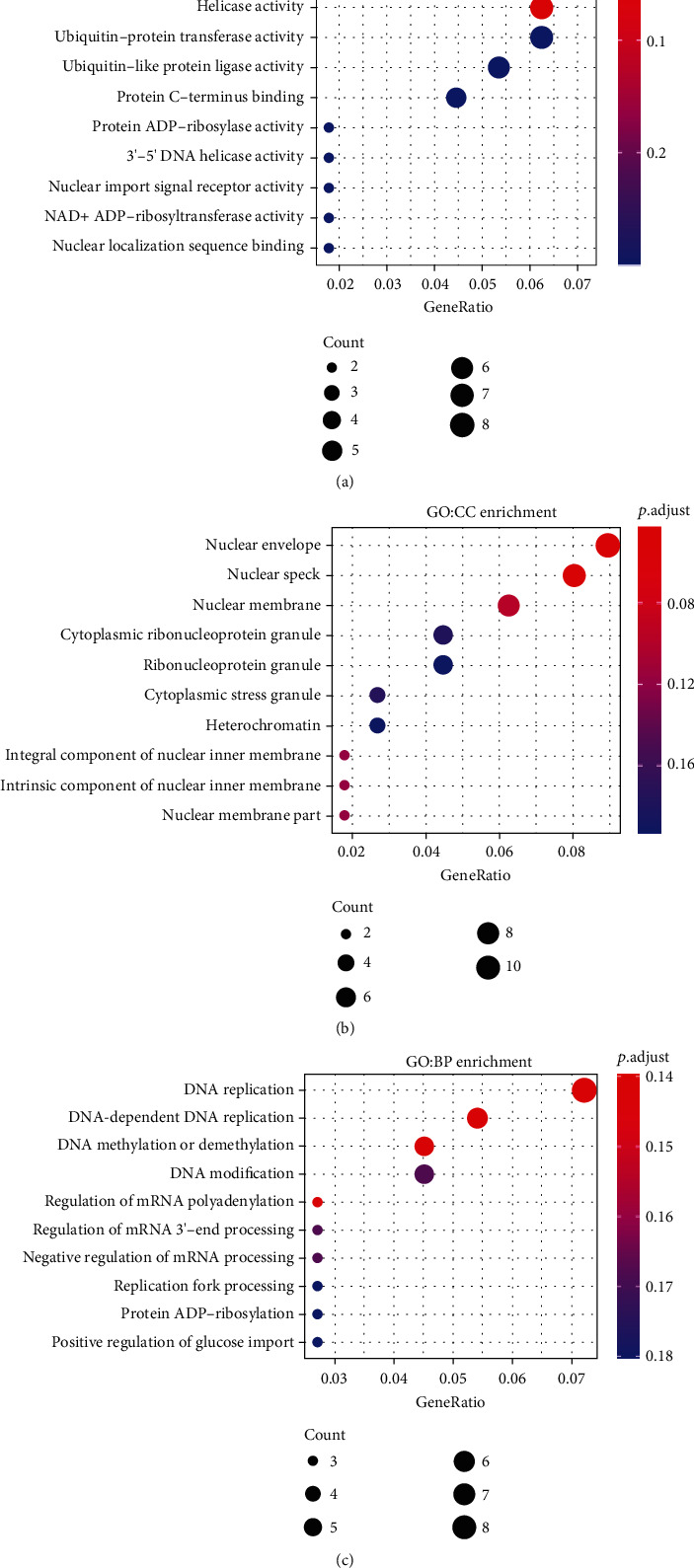
GO pathway enrichment of key module genes. (a)–(c) A, B, and C are the results of GO enrichment analysis at molecular, cellular, and pathway levels, respectively.

517 genes, including FTO and RFX7, could be identified using differential gene analysis of expression patterns from clusters A and B. The default 1 percent difference gene screening criteria are used, followed by *p* < 0.01 and an absolute logFC ≥ 0.5. The differentially expressed genes were built using WGCNA. Two modules (Figure 4(a)), Blue and Turquoise, were built with the optimum soft value of 20. These two modules have a strong connection to the characteristics of clusters A and B (Figure 4(b)). The WGCNA modules were subjected to GO and KEGG pathway enrichment analyses. Figure 3(c) shows that the Blue module is strongly associated with clusters A and B (*p* < 0.0001), and therefore, the GO and KEGG findings will be shown on the Blue module. However, the KEGG database was unable to enrich Blue module genes; thus, only GO database enrichment findings at the molecular, cellular, and pathway levels were shown in the end. According to the findings, the Blue Module genes may be more abundant in enzymes like ubiquitin-like protein transferase and helicase (Figure 5(a)). The module genes may be enhanced in the nuclear envelope at the cellular level (Figure 5(b)). DNA replication may be enhanced in vast pathways thanks to these gene modules (Figure 5(c)). This means that these genes are crucial for cellular biology.

### 3.6. Hub Gene Network of PPI

**Figure 6:**
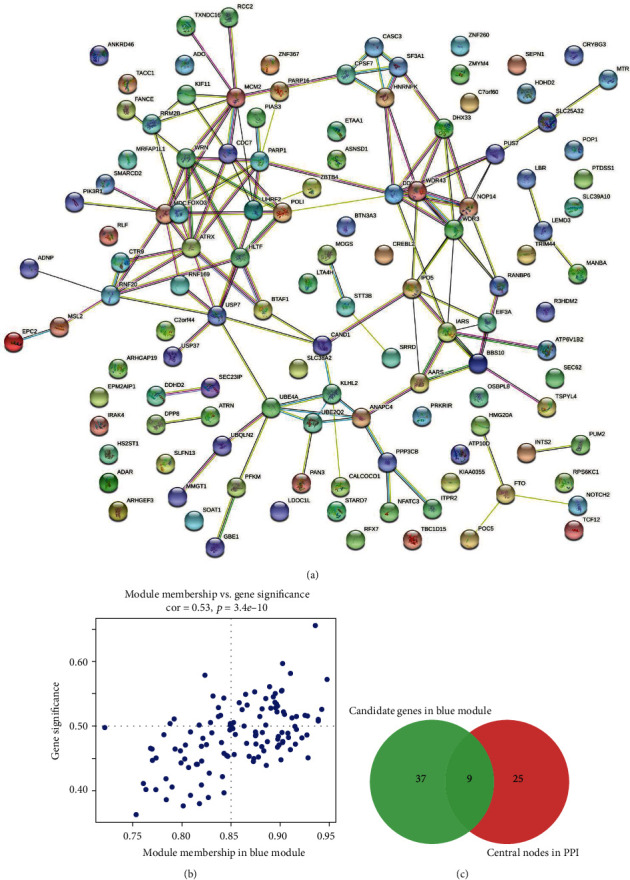
Hub gene network diagram of PPI interactive network screening. (a) PPI interaction network diagram. (b) Correlation analysis between Blue module and gene importance in WGCNA. (c) PPI network and WGCNA two methods to screen the number of hub genes.

121 genes such as FTO, RFX7, and NFATC3 were extracted from the Blue module.  Figure 6(a) shows how the STRING website's PPI was generated for these genes. Cytoscape's cytoHubba plug-in was used to extract the hub genes. The Blue module of WGCNA contains 121 genes, and we isolated and tested 46 hub genes using the following screening conditions: clusters A and B have a correlation coefficient of more than 0.5, and Blue module has a correlation coefficient of more than 0.8, according to the results of the correlation study (Figure 6(b)). Final screening included RRM2B, CASC3, SF3A1, IPO5, BBS10, ATRX, and WRN genes as well as PARP1 and RANBP6 hub genes (Figure 6(c)).

### 3.7. Heterogeneity of Hub Genes among m6A-Cluster Subtypes

**Figure 7:**
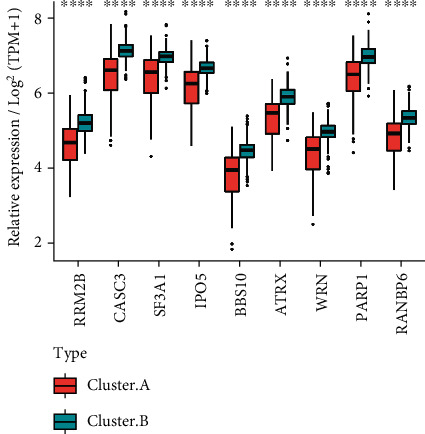
The different expression levels of hub gene among m6A-cluster subtypes. Hub gene was differentially expressed among m6A-cluster subtypes. ^∗∗∗∗^*p* < 0.0001.

**Figure 8:**
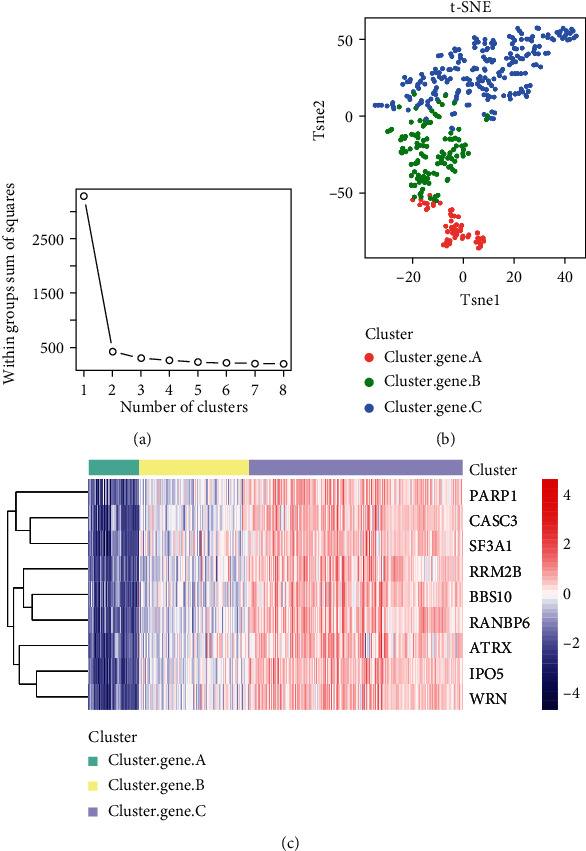
Molecular types on the samples by hub genes. (a) Sum of squares showed samples had the best effect when they were divided into three groups by *K*-means clustering. (b) *t*-SNE dimension reduction analysis. (c) Hub genes expression patterns of three groups in UC.

9 hub genes' levels of expression were examined between clusters A and B. These 9 genes had substantially different levels of expression in the two groups, with all of them being considerably lower in samples from cluster A than cluster B. (Figure 7). All of the UC samples were subjected to *K*-means clustering to learn more about hub gene expression patterns in UC. *t*-SNE dimension reduction analysis revealed the expression patterns of nine genes in the Tsne1 and Tsne2 dimensions that could substantially differentiate the three groups of samples through sum of squares (Figure 8(a)) and A, B, and C (Figure 8(a)) via sum of squares (Figure 8(b)). It was easy to see from the heat map which samples had the most distinct expression patterns for the hub genes in each group. Cluster gene A had the lowest expression levels, cluster gene B had the middle levels, and cluster gene C had the highest levels (Figure 8(c)).

### 3.8. Characterization of m6A Cluster Subtypes and cluster_gene Subtypes of UC

**Figure 9:**
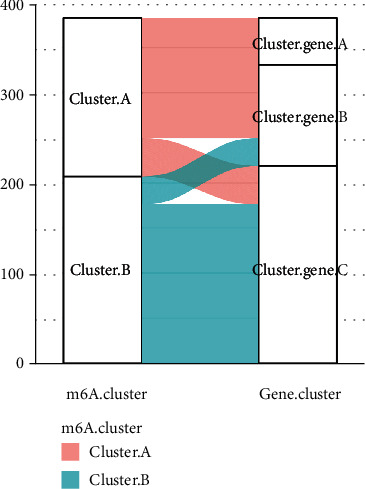
The change from m6A cluster groups to cluster_gene groups. The Sankey diagram showed the change from m6A cluster groups to cluster_gene groups.

**Figure 10:**
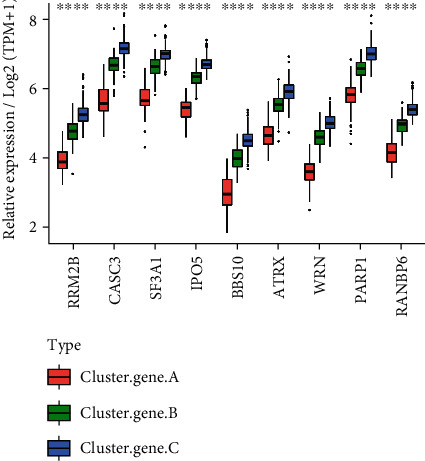
Hub genes differ between cluster_gene groups. Display of hub gene differences between groups. ^∗∗∗∗^*p* < 0.0001.

The Sankey diagram of all UC samples showed the change from m6A cluster groups (cluster A and cluster B) to cluster_gene groups (cluster_gene A, cluster_gene B, and cluster_gene C) grouping (Figure 9). After that, we looked at the disparities in gene cluster groups in terms of hub genes. We discovered that the expression levels of nine hub genes differed substantially across the samples of cluster gene A, cluster gene B, and cluster gene C, with the lowest levels reported in cluster gene A samples by comparing the expression levels of the nine hub genes. In the cluster gene B group, gene expression was moderate, whereas it was the greatest in the cluster gene C group (Figure 10).

## 4. Discussion

A chronic inflammatory illness of the colon, ulcerative colitis, is becoming more common throughout the globe [17]. Genetic susceptibility, epithelial barrier abnormalities, dysregulated immune responses, and environmental variables all play a role [18, 19]. When it comes to biological complexity, understanding the meaning of UC is a must. UC is an example of an inflammatory bowel disease (IBD) that has a high degree of complexity and diversity.

**Figure 11:**
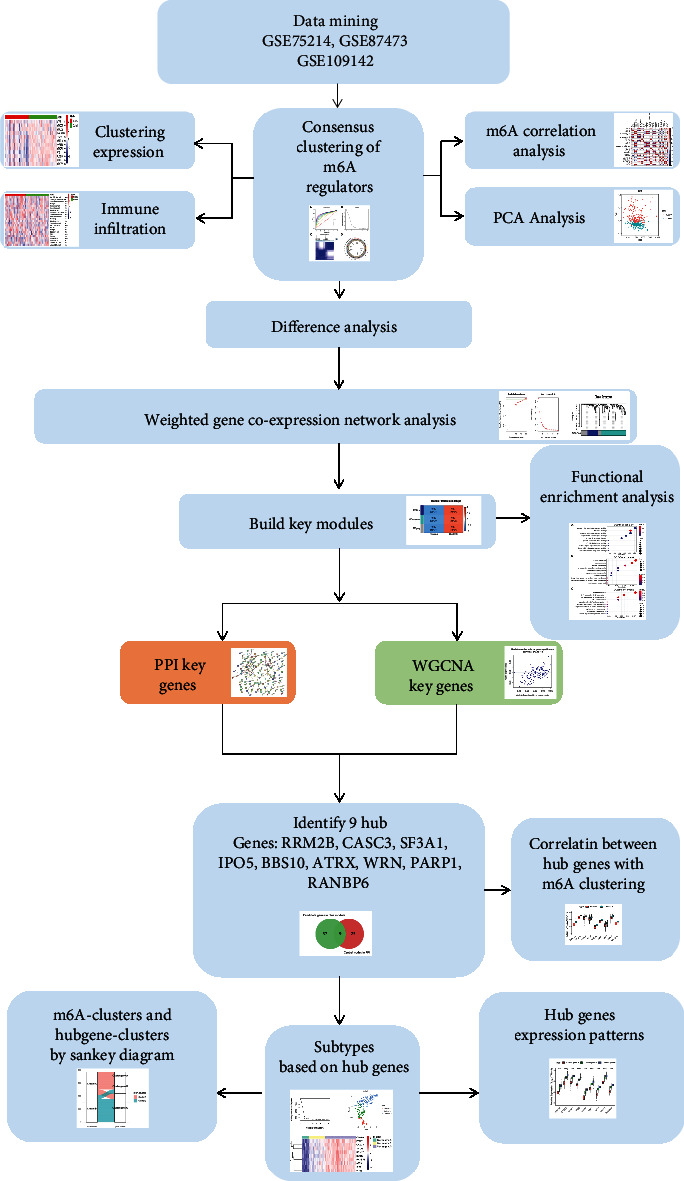
Graphical abstract: flow chart of the study.

Inherently heterogeneous affected people and the surrounding environment contribute to complexity, while a vast number of interacting variables and processes contribute to unpredictability. Because of this, finding and establishing a regulatory analysis that takes into account the variability of UC are critical. The expression of RNA methylation regulators of epigenetic is strongly related to the heterogeneity and prognosis of UC, according to recent study. This is a significant finding. And we draw a graphical abstract to demonstrate the analysis workflows of this study (Figure 11). In this study, starting from the expression of 12 m6A regulators, the expression profile data of GSE75214, GSE87473, and GSE109142 were integrated and analyzed for the next step. Through consensus clustering based on the expression of most aberrant m6A regulators, two m6A molecular subtypes were identified in UC, cluster A/B. And the expression of 12 m6A regulatory genes in cluster A was significantly lower than that in cluster B. In addition, the degree of immune cell infiltration differed somewhat between the two groups. There were also substantial differences in the expression levels of key cytokines between the two groups. These findings suggest that the distinction between clusters A and B is important and warrants additional study. For this reason, the expression profiles of clusters A and B were compared to look for differentially expressed genes, the WGCNA and PPI networks were built to look for such genes, and lastly, nine hub genes were filtered and examined. These hub genes will serve as crucial study targets for us in the future, since they point us in the right path.

Since the discovery of epigenetic changes such as methylation a decade ago, many studies have shown the critical role played by m6A in immune cell homeostasis control and other pathways associated with it. T cell development is adversely affected by m6A because it delays induction of synthesis rate, regulates processing rate, and continuously increases degradation rate. The loss in T cells of METTL14, a subunit of the RNA methyltransferase, induces spontaneous colitis in mice, according to a recent research [6]. In our study, we retyped the UC patients with m6A characteristics, and it is shown that the expression levels of YTHDC1, RBM15, METTL3, HNRNPC, YTHDF1, FTO, ALKBH5, and YTHDF2 were significantly higher in cluster B (*p* < 0.0001). A recent research revealed that YTHDF1 knockout in mice dampened tumor growth in an inflammatory CRC model, and DNA copy number gain of YTHDF1 is a frequent event in CRC and contributes to its overexpression [20]. YTHDF2 silenced in human HCC cells or ablated in mouse hepatocytes provoked inflammation, vascular reconstruction, and metastatic progression [21]. One m6A writer RBM15 and one eraser FTO significantly modulate the expressions of m6A-RMRs, suggesting a feedback mechanism for regulation of m6A-RMR expressions [22]. And there were clearly differences between subtypes among immune cells, such as Th cell, NK cell, monocyte, and macrophage (*p* < 0.0001). The expression levels of cytokines in cluster A and cluster B groups also differed greatly, for example, the expression levels of IL16 and PDCD1 were significantly higher in cluster B than in cluster A. The previous studies identified RBM15 and METTL3 as m6A writers, HNRNPC, YTHDF1, and YTHDF2 as m6A readers, and ALKBH5 and FTO as m6A erasers. In order to exercise its effects, m6A recruits proteins that bind to m6A (m6A readers) to specific RNAs. S-Adenosylmethionine (SAM) transferase then uses methyl groups to install the m6A alteration on the targeted RNAs through the nuclear speckle's m6A writers, which are composed of an enzyme called m6A writers and m6A methyltransferase [23]. The discovery of demethylases (erasers) that remove methyl groups from m6A shows that this RNA alteration is dynamically reversible. Because of Writers and Erasers' bidirectional modulation impact, researchers now have a better clinical knowledge of the illness, and future studies to assist clinical diagnosis and therapy will likely investigate additional m6A modification-related consequences.

To further explore the underlying molecular mechanisms and progress, we utilized WGCNA and PPI. 121 genes such as FTO, RFX7, and NFATC3, were extracted from the Blue module. PPI was constructed for these genes on the STRING website. We extracted and screened 46 hub genes from 121 genes in Blue module of WGCNA, by integrating all the data, RRM2B, CASC3, SF3A1, IPO5, BBS10, ATRX, WRN, PARP1, and RANBP6 hub genes were screening out. The overview of all hub genes, including the function and introduction, was shown in Table 2. It is reported that patients with UC have a significant risk of developing CRC [24]. Despite modern screening procedures, only approximately half ulcerative colitis-related colorectal cancer (UC-CRC) patients are diagnosed at the advanced stage and have a poor prognosis. For example, in a past study, RRM2B would be an eligible prognostic biomarker to predict survival and therapeutic response for in advanced CRCs. In the clinical specimens study, researchers found that RRM2B significantly related to better overall survival in stage IV CRC patients (HR = 0.40, 95% CI 0.18–0.86, *p* = 0.016) of the training set [25]. This study revealed that RRM2B play a crucial role in CRC.

In conclusion, further exploration of m6A subtypes will help us to better understand and investigate the processes of UC and clarify the relationship between inflammation and tumor growth and progression. Given the molecular complexity of UC and the individual uniqueness and unpredictability of individuals, it is important for the creation of tailored therapy. The discovery of 9 potential genes that are controlled by m6A methylation-dependent pathways raises the possibility that m6A methylation plays a role in the etiology of ulcerative colitis. A total of 9 potential genes may prove to be valuable indicators for the severity of UC illness and the development of novel focused therapy techniques for UC patients.

## Figures and Tables

**Table 1:** Information on the 12 m6A RNA methylation regulators.

Gene symbol	Abbreviations	Types
Vir-like m6A methyltransferase-associated (VIRMA, also called KIAA1429)	KIAA1429	m6A writers
Methyltransferase-like 3	METTL3	m6A writers
Methyltransferase-like 14	METTL14	m6A writers
RNA binding motif protein 15	RBM15	m6A writers
Wilms tumor 1-associated protein	WTAP	m6A writers
Heterogeneous nuclear ribonucleoprotein C	HNRNPC	m6A readers
YTH domain containing 1	YTHDC1	m6A readers
YTH domain containing 2	YTHDC2	m6A readers
YTH N6-methyladenosine RNA binding protein 1	YTHDF1	m6A readers
YTH N6-methyladenosine RNA binding protein 2	YTHDF2	m6A readers
AlkB homolog 5	ALKBH5	m6A erasers
Fat mass and obesity-associated protein	FTO	m6A erasers

**Table 2:** Information on the 9 hub genes.

Gene symbol	Abbreviations	Function
Ribonucleotide reductase M2B	RRM2B	p53R2 is a subunit of the ribonucleotide reductase complex [26].
Cancer susceptibility candidate 3	CASC3	As a core component of the exon junction complex (EJC), CASC3 was described to be pivotal for EJC-dependent nuclear and cytoplasmic processes [27].
Splicing factor 3A subunit 1	SF3A1	SF3A1 is a protein coding gene [28].
Importin-5	IPO5	A member of the karyopherin beta subunit locates in the 13q32 chromosomal region and has been demonstrated to play a vital role in the translocation of various proteins [29].
Bardet-Biedl syndrome 10	BBS10	Among its related pathways are cargo trafficking to the periciliary membrane and organelle biogenesis and maintenance [30].
Alpha thalassemia/mental retardation, X-linked	ATRX	ATRX is a chromatin remodeling protein whose main function is the deposition of the histone variant H3.3 [31].
Werner's syndrome gene	WRN	A member of the RECQ family of DNA helicases plays essential roles at stalled forks to counteract replication stress, thereby ensuring genomic stability [32–34].
Poly(ADP-ribose) polymerase 1	PARP1	Upon activation, PARP1 attaches ADP-ribose polymer chains to target proteins using nicotinamideadenine dinucleotide (NAD+) as a substrate and facilitates the process of DNA repair [35].
Ran-binding protein 6	RANBP6	As member of the importin *β* superfamily and EGFR regulator [36].

## Data Availability

The original contributions presented in the study are included in the article. Further inquiries can be directed to the corresponding author.
